# Ankle-foot orthosis mechanical testing: a scoping review

**DOI:** 10.1186/s12984-026-02072-w

**Published:** 2026-06-30

**Authors:** Katherine Vaiciulis, Zachary Williston, Deema Totah

**Affiliations:** Department of Mechanical Engineering, University of Iowa, 3131 Seamans Center, Iowa City, IA 52242 USA

**Keywords:** Ankle-foot orthosis, AFO, Stiffness, Resistance, Compliance, Rigidity, Mechanical testing

## Abstract

**Background:**

Ankle-foot orthoses (AFOs) are assistive devices that support the ankle during gait. AFO mechanical properties, such as rotational stiffness about the ankle, influence the level of support these devices provide. Interest in quantifying AFO mechanical properties is on the rise but is limited by the lack of standardized measurement and assessment techniques. This scoping review discusses existing techniques for the measurement of AFO mechanical properties and introduces a taxonomy for organizing and describing these methods.

**Methods:**

A scoping review was conducted using a snowballing approach starting from two previously published literature reviews and a database search using PubMed, Engineering Village (Compendex), and Web of Science. Articles with methods that experimentally measured the mechanical properties of AFOs were eligible for inclusion. Data extracted from the selected articles included operation specifications, planes/joints tested, and presence of a biological or surrogate limb during AFO testing.

**Discussion:**

The majority of methods utilize benchtop AFO testing, with a few in-situ methods that involve a biological limb. We highlight key design challenges related to replicating biological gait, including bending axis location and design, motion in single versus multiple planes, and limited testing speeds and range of motion.

**Conclusion:**

We introduced a taxonomy for describing AFO testing methods to facilitate cross-device comparisons and inform future development. Future work should include developing standardized, biomimetic methods and validation techniques for testing a variety of AFO designs. This would enable more accurate AFO measurement and enable cross-study comparisons, ultimately leading to optimized AFO properties and improved user performance outcomes.

## Introduction

**Fig. 1:**
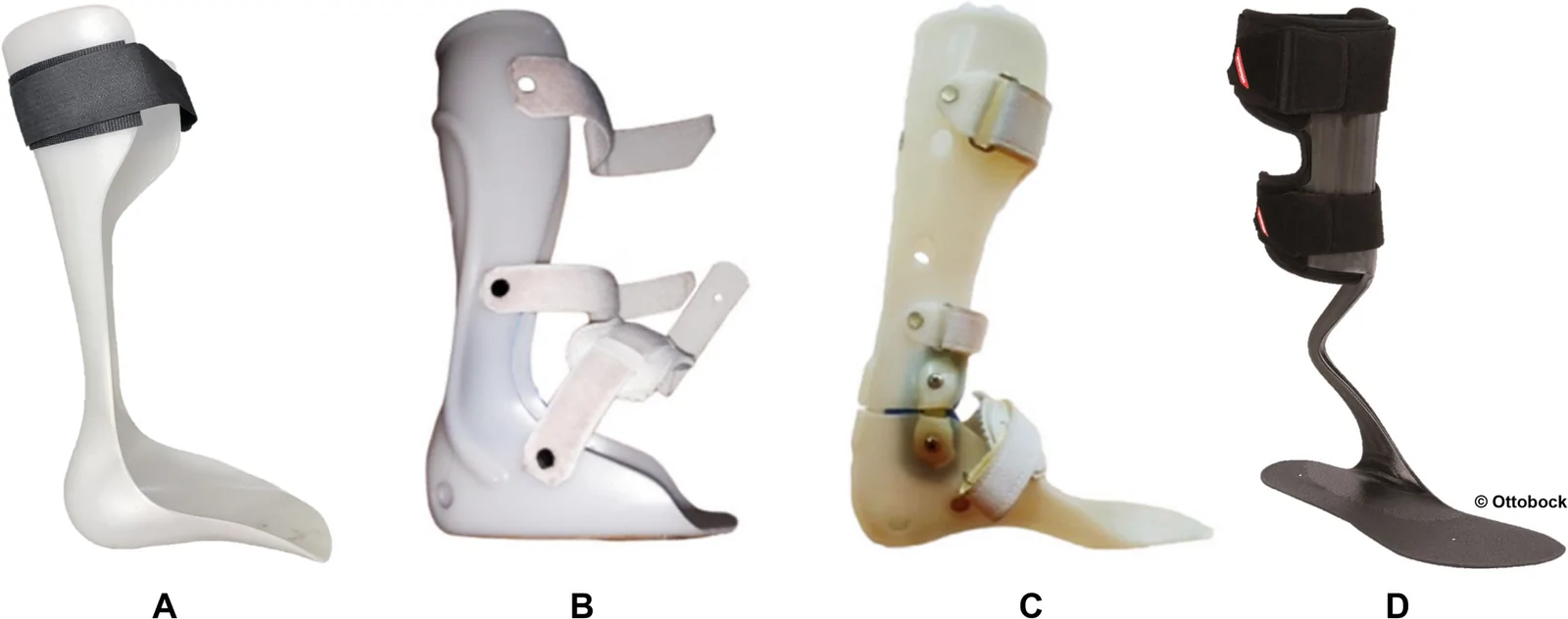
Sample AFOs. **A** Thermoplastic posterior leaf spring AFO. **B** Solid AFO, adapted from [7], licensed under CC BY 4.0 (https://creativecommons.org/licenses/by/4.0/). **C** Hinged AFO, adapted from [7], licensed under CC BY 4.0 (https://creativecommons.org/licenses/by/4.0/). **D** Carbon fiber anterior AFO, reproduced from [8], with permission

Ankle-foot orthoses (AFOs) are externally applied assistive devices worn around the ankle joint. They are commonly used among individuals with neurological and/or musculoskeletal conditions such as cerebral palsy [1], Charcot-Marie-Tooth [2], or multiple sclerosis [3], and for rehabilitation post-stroke [4]. AFOs are often intended to support the ankle during gait, but their functional requirements vary between users and are influenced by material selection and design (Fig. 1). For some, they alleviate pressure or pain from deformities. For others, AFOs modify the joint range of motion. A frequent cohort of AFO users are individuals experiencing drop-foot, where dorsiflexor muscle weakness causes an individual’s toes to point downward and drag during the swing phase of gait. This inability to achieve toe clearance increases the risk of trips and falls. An AFO can alleviate this by keeping the forefoot lifted during the swing phase to achieve foot clearance.

The modern thermoplastic AFO (Fig. 1) was developed in 1971, followed by the first efforts to measure AFO properties in 1977 [5]. This approach modeled jointed AFOs as two rigid bodies linked by a fulcrum lever and a toe-raising spring. This spring represented the AFO’s mechanical properties. As the AFO is moved into plantarflexion, a resistive dosiflexing moment is created that is proportional to this modeled spring stiffness. The greater the stiffness, the greater the moment experienced. Similar models are still used today and have been applied across a range of AFO designs, often representing AFO compliance or stiffness as a rotational spring about the ankle joint, with some modeling resistance to plantarflexion and dorsiflexion separately [6]. Measures of AFO stiffness are typically represented using a torque-angle curve, displaying the torque required to flex the AFO to specified angles. The stiffness parameter is then calculated as the slope of a linear fit to portions of the torque-angle curve (see Sect. 2.2).

**Fig. 2:**
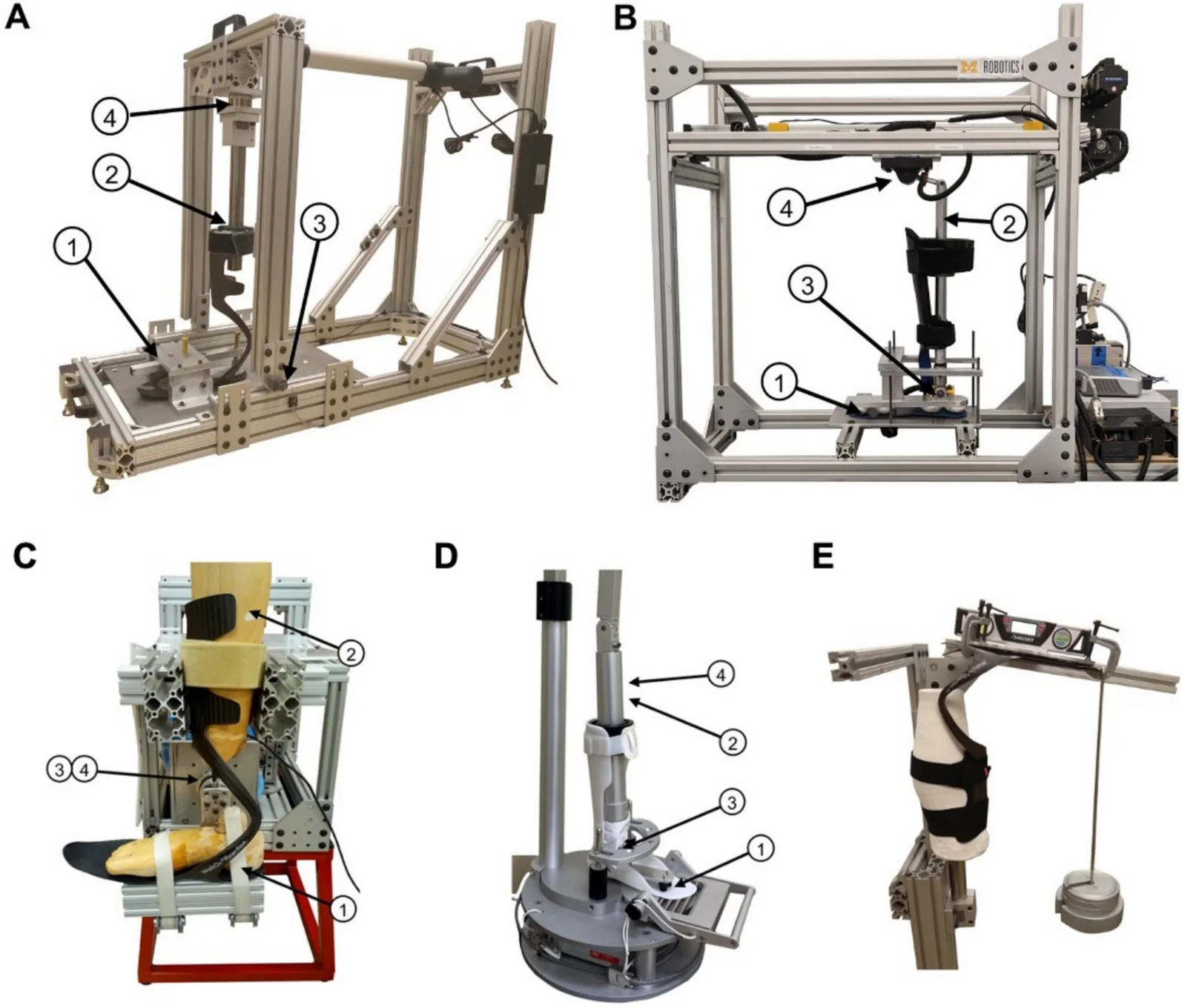
Five testing methods used in a comparison of AFO stiffness measurement methods, adapted from [9], licensed under CC BY 4.0 (http://creativecommons.org/licenses/by/4.0/). The dynamic testing methods (**A-D**) all consist of (1) fixing the AFO footplate to a surrogate foot, (2) fixing the AFO calf band to a surrogate shank, rotating the AFO and measuring the resulting (3) angular displacement and (4) applied force. The static testing method (**E**) consists of hanging weights from the AFO footplate and measuring the resulting deflection angle. **A** Evaluating Mechanical Properties In Rotating Exoskeletons (EMPIRE), **B** Stiffness Measurement Apparatus (SMApp), **C** Kentucky Stiffness Tester (KST), **D** Bi-articular Reciprocating Universal Compliance Estimator (BRUCE), **E** Hanging weight method

Recent studies have found that individually optimizing AFO stiffness improves gait performance [10–14]. However, quantitative methods for individual AFO optimization remain largely confined to research settings and are not commonly used in clinical practice. Existing AFO prescription resources are mostly qualitative, including policies for determining medical necessity [15], guidance for choosing between AFO designs [16], and condition-specific recommendations for individuals with stroke [17] or cerebral palsy [1].

In practice, clinicians typically use observational gait assessment to guide AFO prescription decisions [18]. This approach often requires iterative trial-and-error adjustments, relying heavily on clinician expertise and experience to evaluate an individual while walking with and without an AFO. Numerous studies have shown that AFOs benefit users by improving gait parameters [19, 20]. However, the lack of standardized, quantitative prescription protocols leads to varied practices between clinicians [18], which may contribute to inconsistent user outcomes. For some individuals, AFOs do not improve certain gait or motor function parameters [21–24], highlighting the variability in user response and suggesting that AFO benefits are not universal. These findings underscore the need for a quantitatively-informed, individualized AFO prescription process to ensure optimal treatment. An essential part of this process must be the accurate measurement of AFO stiffness.

Some research groups have proposed frameworks for incorporating individually optimized AFO stiffness into the prescription process. For example, one protocol uses a selection algorithm based on walking energy cost, speed, and clinical assessment of gait biomechanics while an individual walks with five different AFO stiffness levels [25]. Another proposed framework consists of a systematic, evidence-guided algorithm to adjust AFO stiffness based on ankle and knee kinematics during key points of the gait cycle [26]. Using frameworks like these to incorporate AFO stiffness measurement into clinical decision-making has the potential to address current clinical challenges and transform the field. By providing an objective, evidence-based protocol, the clinical process would be significantly streamlined while optimizing user outcomes.

Interest in AFO mechanical properties is on the rise, evident by the increasing number of studies measuring AFO stiffness and its effects on user outcomes in recent years. A comprehensive literature review published in 2019 includes 28 articles that experimentally evaluate AFO mechanical properties, alongside additional papers focused on computational analysis of AFO properties [27]. Although several research groups have developed techniques for AFO stiffness measurement, validating testing methods is difficult and differences in measurements across methods have been observed. A recent experimental study measured the stiffness of 13 AFOs using five different testing methods and found significant differences in measured stiffness between methods [9]. The experiment used four previously published, dynamic benchtop testing methods and one simpler, static loading method (Fig. 2). The median relative percent difference in measured AFO stiffness between testing methods was 62%. When the static loading method was excluded, the median relative percent difference was 27% across the four dynamic testing methods [9]. Potential sources of variation include differences in the clamping mechanism securing the AFO during testing, axis of rotation location, flexion speed, and surrogate leg design across methods.

Overall, the variability in measured AFO stiffness highlights the need for standardization of AFO testing methods and terminology to describe and categorize differences between methods [6]. There is no standard classification or agreed upon terminology for reporting these differences, making it difficult to compare methods. With the advancement of smart materials and manufacturing methods like additive manufacturing, there is an anticipated expansion of the design space for devices like AFOs, so it is crucial to understand the current landscape of AFO evaluation.

In this scoping review, we highlight a comprehensive selection of 46 papers conducting experimental testing of AFO properties that represent the field. We provide an overview and analysis of existing measurement methods for evaluating AFO mechanical properties, with a focus on rotational stiffness about the ankle, and introduce a taxonomy based on their properties to organize different methods. This paper begins with an explanation of the ankle joint’s motion during gait, providing context for the joint that benchtop testing methods aim to replicate (Sect. 2.1), and an introduction to modeling AFO stiffness (Sect. 2.2). Next, we outline our search strategy (Sect. 3) and provide a detailed breakdown of key design considerations for AFO testing methods, along with suggested terminology and a framework for organizing testing methods (Sect. 4). Finally, strengths and limitations of existing methods are identified, highlighting key challenges in AFO mechanical testing and suggestions for future directions (Sect. 5).

## Background

### Ankle motion during the gait cycle

**Fig. 3:**
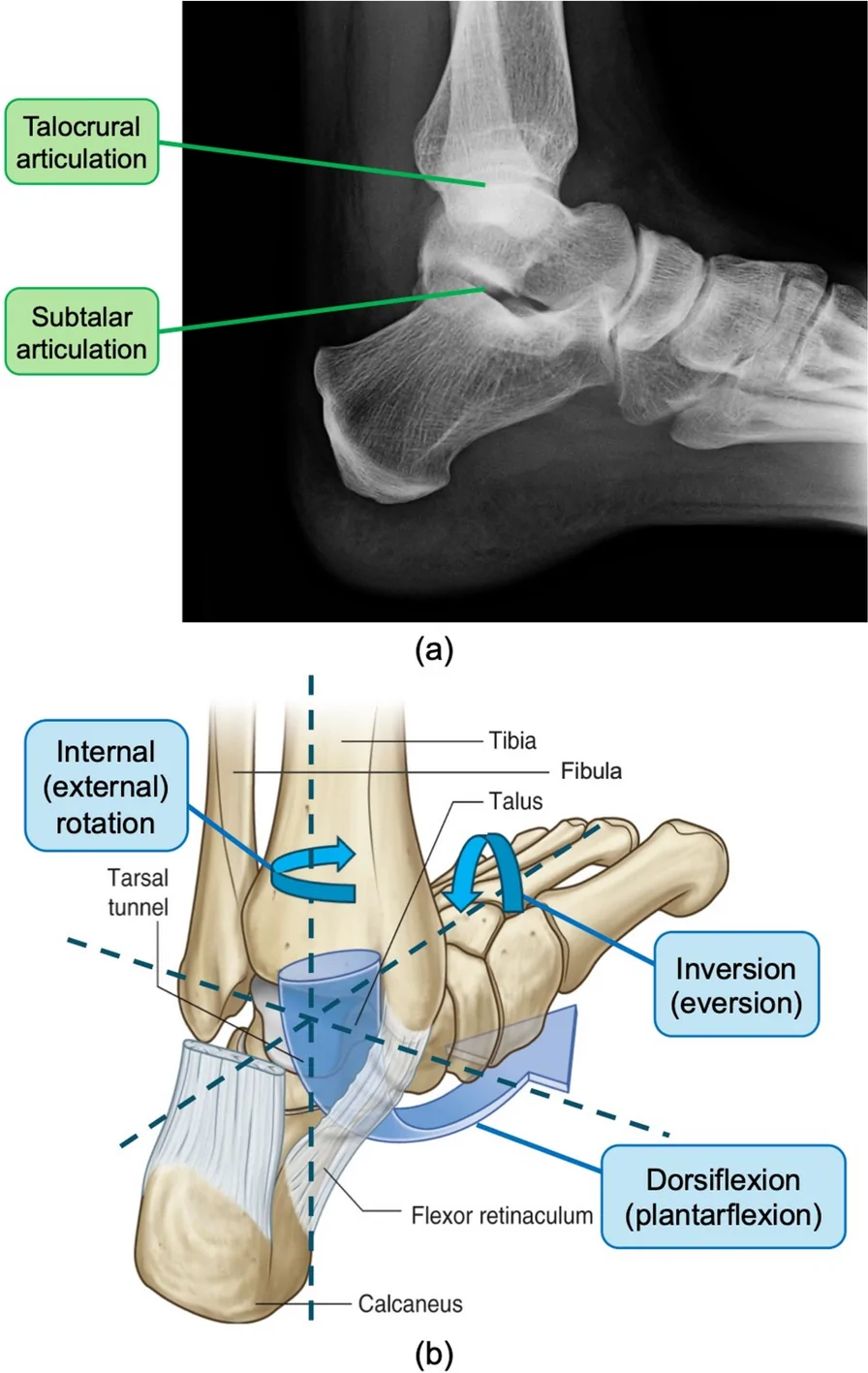
(a) Articulations of the ankle joint complex. Adapted from [28], licensed under CC BY-SA 2.0 (https://creativecommons.org/licenses/by-sa/2.0/). (b) The bony anatomy and motions of the ankle joint complex, motions are labeled in boxes. Adapted from [29] with permission from Elsevier

Because AFO testing methods aim to measure AFO properties experienced by the user, they attempt to replicate or mimic biological ankle motion. Thus, it is important to understand the biomechanics of the ankle joint during gait. The ankle joint complex is the site where the fibula, tibia, talus, and calcaneus meet. It is comprised of two main articulations: the talocrural joint and the subtalar joint (Fig. 3a). The talocrural joint is the proximal portion, where the tibia and fibula articulate with the talus. The subtalar joint is the distal portion, where the talus articulates with the calcaneus. These compounding joints work together to rotate the ankle joint complex in three degrees of freedom: dorsiflexion/plantarflexion, inversion/eversion, and internal/external rotation (Fig. 3b). We will focus on the biomechanics of the ankle joint during the stance phase of gait, as this is the weight-bearing portion when a user applies a load to their AFO.

**Fig. 4:**
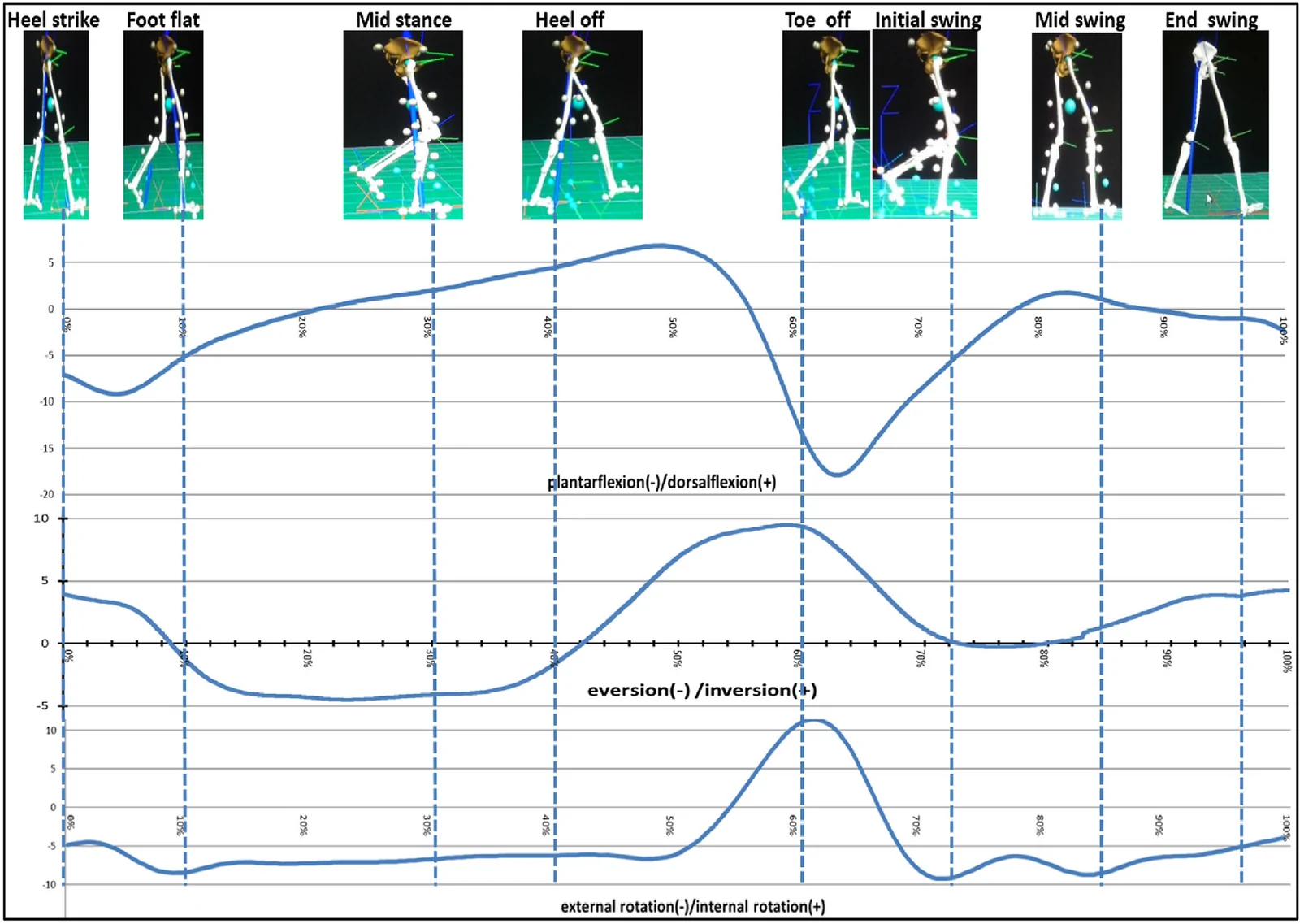
Three-dimensional ankle kinematics during the gait cycle, reproduced from [30], licensed under CC BY-NC-ND 4.0 (https://creativecommons.org/licenses/by-nc-nd/4.0/). The horizontal axis is the normalized gait cycle from 0 to 100%, and the vertical axis is ankle joint angles

Throughout the gait cycle, the ankle undergoes rotations in all three degrees of freedom (Fig. 4). The main motion of the ankle joint complex involves cycles of dorsiflexion and plantarflexion at the talocrural joint. However, this motion does not act like a pure hinge joint due to the non-uniform curvature of the talus. During weight-bearing dorsiflexion, the talus glides in the posteromedial direction relative to the tibia, and the tibia/fibula rotate internally [31]. During weight-bearing plantarflexion, the talus glides in the anterolateral direction relative to the tibia, and the tibia/fibula rotate externally [31].

Subtalar joint motion is clinically classified as inversion and eversion [32], but weight bearing motion at this joint during gait is more complex. From heel strike to midstance, subtalar joint motion consists of calcaneal eversion and posteromedial glide of the talus [31]. From midstance to preswing, subtalar joint motion consists of calcaneal inversion and anterolateral glide of the talus [31]. These compounding motions of the talocrural and subtalar joints result in a dynamically shifting axis of rotation throughout the gait cycle.

Although ankle motion during the gait cycle is a complex combination of motions in three planes at two joints, it is often conceptualized as simple dorsiflexion and plantarflexion at a hinge joint in the sagittal plane. Therefore, an overwhelming majority of AFO stiffness measurement methods use simplified models of pure dorsiflexion and plantarflexion, neglecting out-of-plane motions. However, AFOs play multiple roles in constraining and supporting the ankle in the different planes of motion. For example, people with reduced lateral stability use AFOs that are rigid in the frontal plane.

### AFO modeling and representations of stiffness

As introduced in the previous section, AFOs are often depicted as multi-body rigid models with motion in the sagittal plane only. AFO stiffness, compliance, or rigidity has been reported in different metrics depending on the model that research groups use to represent the AFO [6]. AFO stiffness has been reported as the overall resistance to motion in plantarflexion only, dorsiflexion only, or both directions. It has also been reported as the stiffness of the AFO’s isolated elastic components or posterior strut. The metrics that have been used to convey AFO stiffness include linear stiffness with units of N/mm, rotational or torsional stiffness (Nm/degree), specific torsional stiffness (Nm/degree/kg), or pressure (kPa); all of which correspond to different models and measurement methods.

**Fig. 5:**
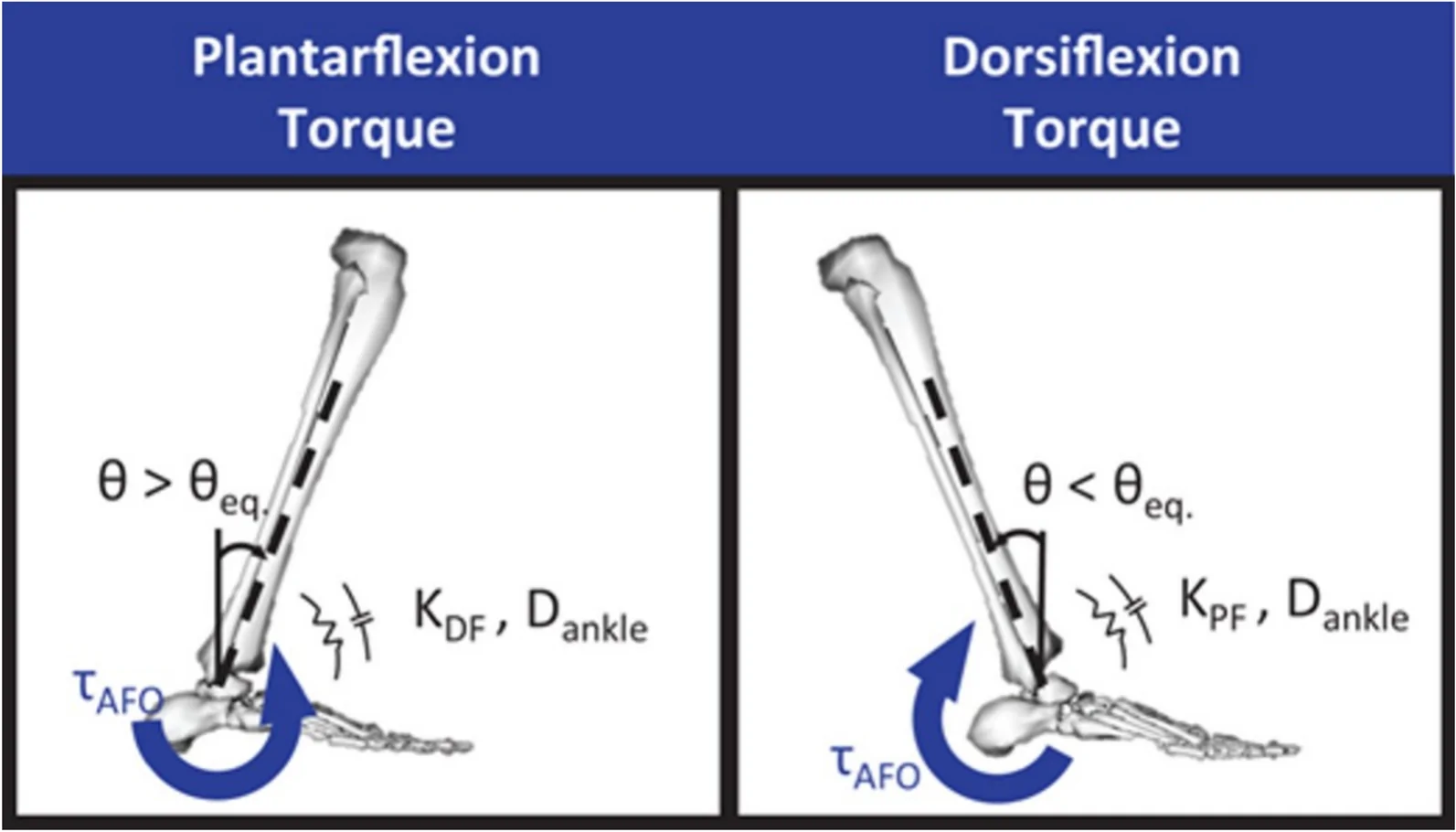
AFO modeled as a spring-damper system with directional stiffness, used with permission of American Society of Mechanical Engineers, from [35]; permission conveyed through Copyright Clearance Center, Inc. $\tau _{AFO}$ is the ankle joint torque, *θ * is the ankle joint angle where $\theta _{eq}$ is the equilibrium angle, K is the directional torsional stiffness in dorsiflexion (DF) or plantarflexion (PF), and $D_{ankle}$ is the damping coefficient

One method of modeling AFOs is a **linear spring**, where the stiffness of the posterior strut equals the slope of the linear fit of the applied force and linear displacement. This stiffness can be calculated through a three-point-bend test, where the linear deflection of the posterior strut is measured in response to a known load [33, 34]. Some researchers have expanded on this to model AFOs as a **spring-damper system** (Fig. 5). In this representation, an AFO is modeled as a “passive torque actuator” with directional stiffness and damping in both dorsiflexion and plantarflexion [35].

**Fig. 6:**
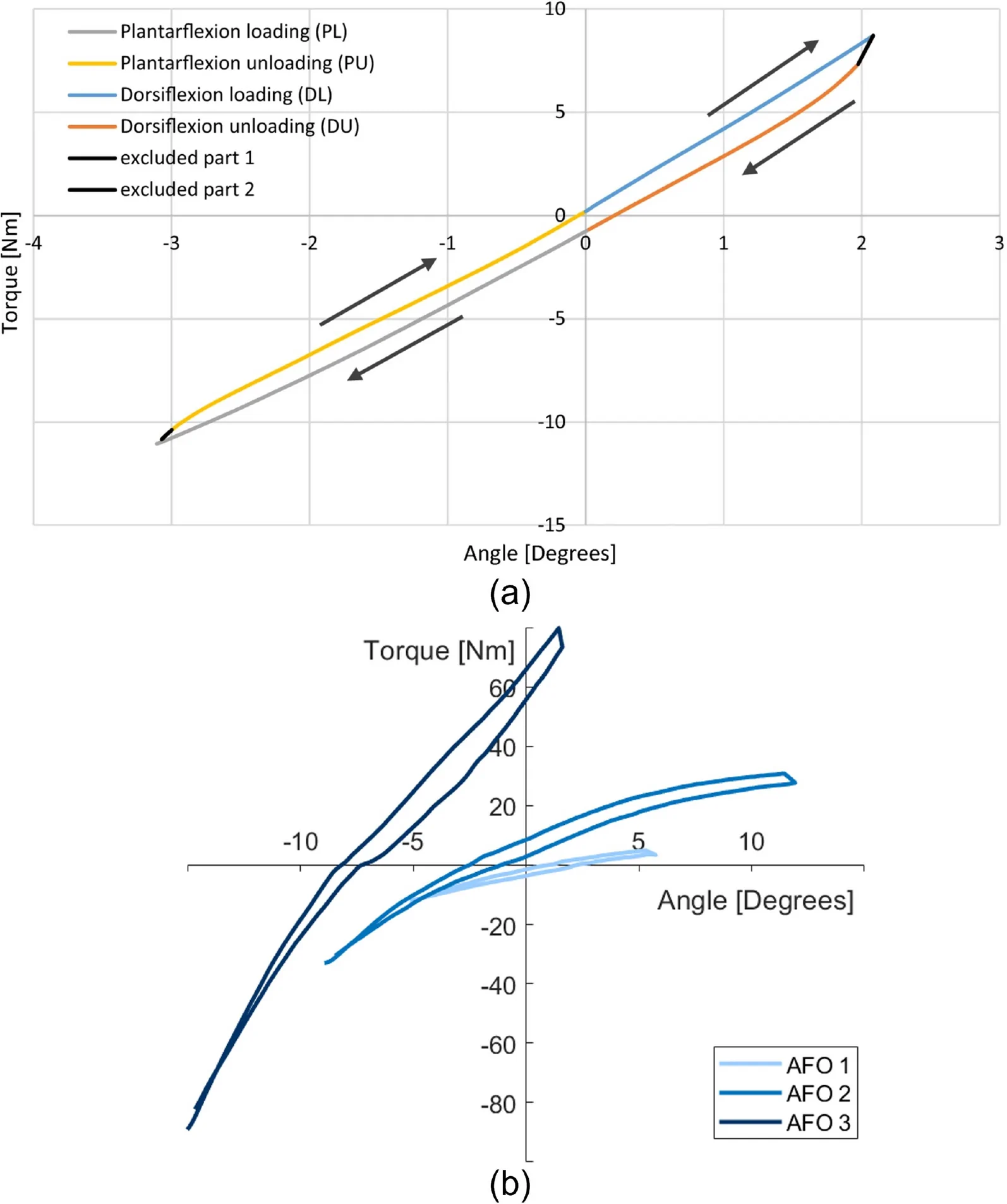
**a** Sample torque-angle curve for AFO stiffness calculation using a piecewise linear model, reproduced from [37], licensed under CC BY 4.0 (https://creativecommons.org/licenses/by/4.0/). **b** Sample torque-angle curves for three AFOs depicting varying levels of stiffness and hysteresis. Curves generated using data from [39]. AFO 1 is a thermoplastic (polypropylene) off-the-shelf AFO with a posterior leaf spring design (weight 125 g, wall thickness 0.2 cm). AFO 2 (305 g, 0.3 cm) and AFO 3 (550 g, 0.5 cm) are solid-ankle AFOs made by fused deposition modeling with carbon fiber enforced nylon

The most common AFO model is a **torsional spring**, where the rotational stiffness equals the slope of the linear fit of the applied torque and angular displacement about the ankle (6a). However, many AFOs have non-linear stiffness or exhibit hysteresis, invalidating the linear fit model assumption. Hysteresis represents an AFO’s energy storage and return property, and its amount varies between AFOs (Fig. 6b). Hysteresis causes a nonlinear torque-angle curve, affecting the slope, and ultimately, AFO stiffness. To address this, some research groups use a bi-linear, or piecewise linear model [36–38], where the torque-angle curve is separated into loading and unloading portions (Fig. 6a). Then, the slopes (stiffness values) of the loading portions during dorsiflexion and plantarflexion are reported separately.

## Methods

This article presents a scoping review using a database search following the Preferred Reporting Items for Systematic reviews and Meta-Analyses extension for Scoping Reviews (PRISMA-ScR) guidelines [40] and a snowballing search [41].

### Search strategy

We conducted a search in PubMed, Engineering Village (Compendex), and Web of Science in August 2025. The keywords used were: (ankle-foot orthosis or AFO or ankle foot orthosis) AND (mechanical properties OR stiffness OR compliance OR rigidity) AND (test OR measure OR evaluate OR quantify). The search was restricted to articles published in English and no limits on publication date were applied. We also conducted a snowballing search using two previously published literature reviews on testing AFO properties as a starting point [27, 42]. We screened the reference list of each of those reviews as well as other papers that cited the reviews. Relevant papers were included in our scoping review and analyzed using the snowballing approach, until no new papers were found.

### Selection criteria

Articles were selected if they met any of the following inclusion criteria:

- Articles with methods that measure AFO properties mechanically (i.e. experimentally)
- Articles that measure AFO properties using existing equipment with or without modifications
- Articles that measure properties of the entire AFO or representative isolated components (e.g., measure posterior strut stiffness)

Exclusion criteria:

- Articles with insufficient details of AFO testing
- Articles that report AFO properties using a previously published testing method
- Articles that measure AFO properties using onboard sensors
- Articles where AFO mechanical properties are not quantified

### Data extraction

From each included article, we extracted the following data: testing operation type, flexion speed, sagittal plane flexion range, ability to accommodate multiple AFO sizes or types, whether reliability testing was conducted, planes and joints tested, and type of limb model used. For articles that did not report information for a given data category, a hyphen (-) was entered in the summary table. The extracted data were synthesized to develop a taxonomy for organizing and describing AFO testing methods, then each included article was classified according to those categories.

### Bias

The authors acknowledge that our background in mechanical engineering may influence the interpretation of the literature, and individuals from different disciplines or areas of expertise may interpret the findings differently. To ensure a comprehensive review, we conducted a systematic search strategy in a variety of databases, covering engineering, medical, and general scientific literature. Additionally, we screened reference lists to ensure the inclusion of relevant papers that did not appear in the database search.

## Results: experimentally quantifying AFO stiffness

**Fig. 7:**
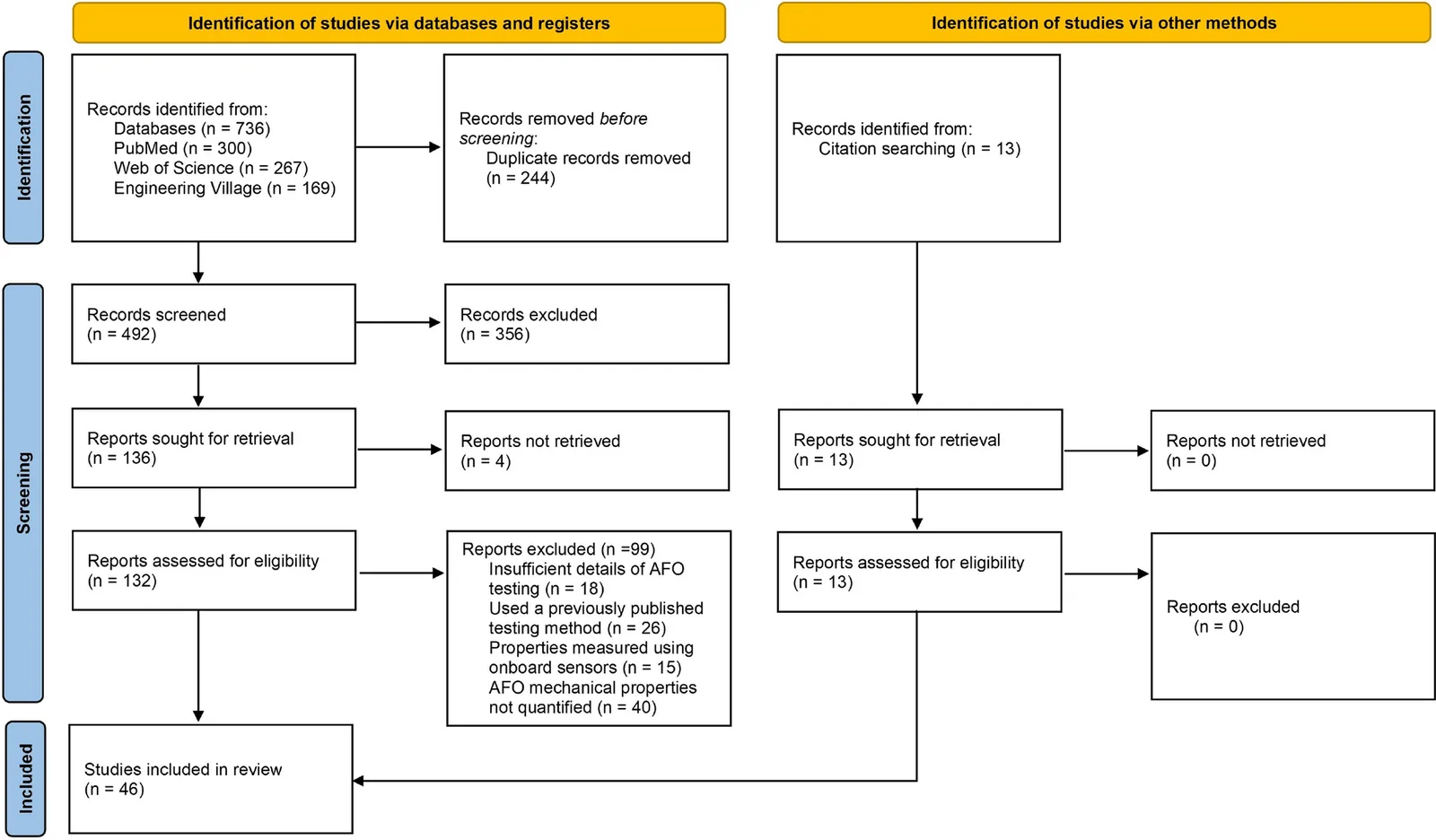
PRISMA flow diagram

**Fig. 8:**
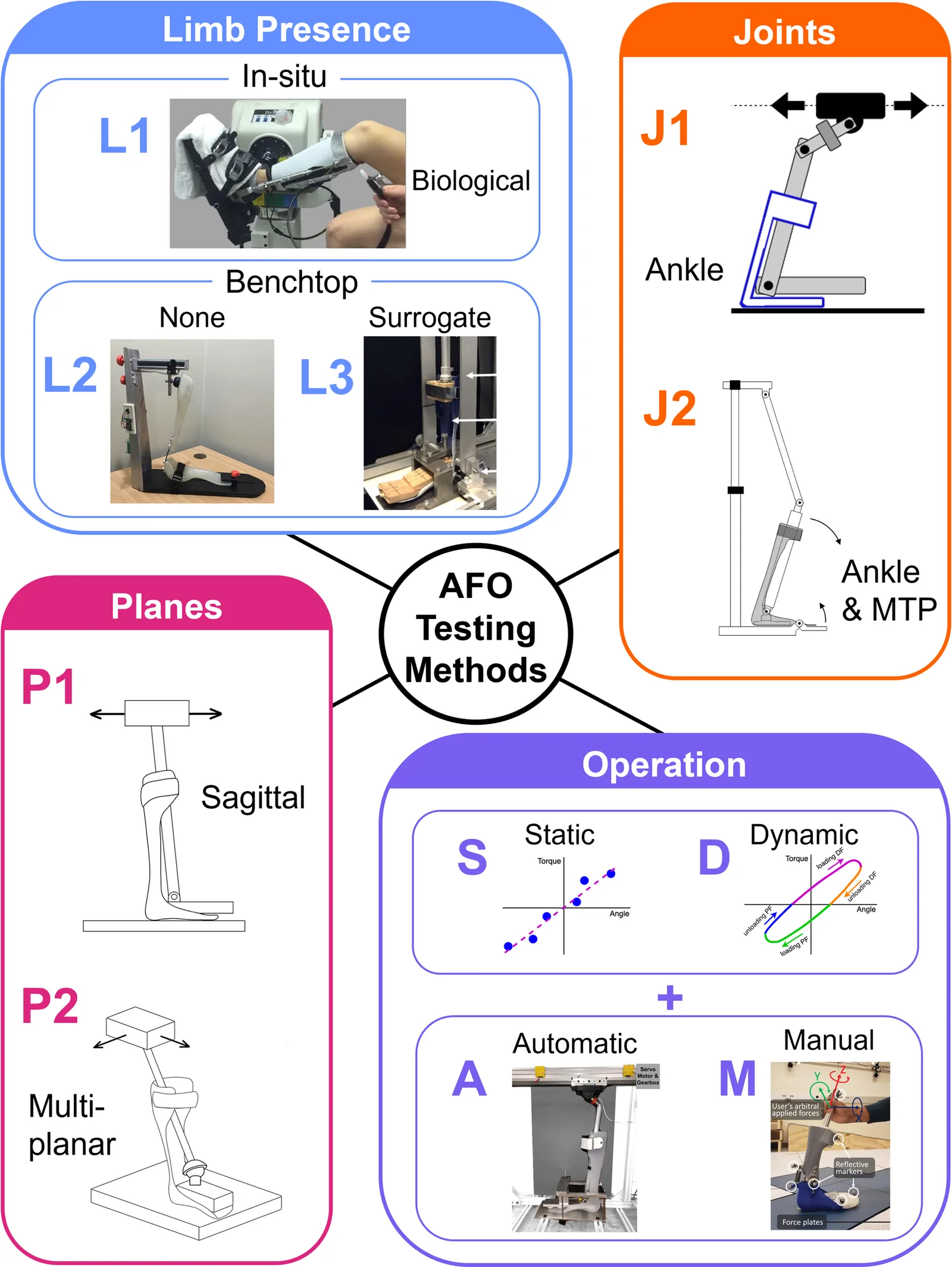
Classifications of AFO testing methods. **L1** Reproduced from [43], licensed under CC BY 4.0 (https://creativecommons.org/licenses/by/4.0/). **L2** Reproduced from [44], licensed under CC BY 4.0 (https://creativecommons.org/licenses/by/4.0/). **L3** Adapted from [37], licensed under CC BY 4.0 (https://creativecommons.org/licenses/by/4.0/). **J1** Original. **J2** Reprinted from [45], with permission from Elsevier. **P1, P2, S, D** Original. **A** Adapted from [36] with permission from Elsevier. **M** Adapted from [46], licensed under CC BY 4.0 (https://creativecommons.org/licenses/by/4.0/)

The database search identified 33 articles for inclusion. By checking reference lists of analyzed articles, 13 additional papers were identified for inclusion. A total of 46 papers were included in this scoping review (Fig. 7) and analyzed to develop categories of AFO testing methods. Based on these categories, we introduce a taxonomy for classifying AFO stiffness measurement methods according to their design and operation properties (Fig. 8). We then classify published works and analyze their testing methods (Table 1). The key testing method categories identified through this search are discussed below in detail.

**Table 1:** Comprehensive selection of AFO testing methods

References	Operation	Flexion speed (deg/s)	Sagittal plane flexion range (deg)	Multi-size/type accommodation	Tested reliability	Classification
Golay et al. [47]	Static	–	0 to 16	–	–	L3–J1–P1–SM
Yamamoto et al. [48]	Dynamic-automatic	10	− 20 to 15	✓	–	L1–J1–P2–DA
Lunsford et al. [49]	Dynamic-automatic	–	− 15 to 10	–	–	L3–J1–P1–DA
Sumiya et al. [50]	Static	–	− 15 to 15	–	–	L3–J1–P1–SM
Nagaya [51]	Static	–	− 12 to 15	✓	–	L2–J1–P1–SM
Klasson et al. [52]	Static	–	− 2 to 3	–	✓	L3–J1–P2–SM
Singerman et al. [53]	Dynamic-manual	–	− 14 to 13	–	–	L3–J1–P1–DM
DeToro [54]	Static	–	− 20 to 20	–	–	L3–J1–P1–SM
Polliack et al. [55]	Static	–	–	✓	–	L3–J1–P1–SM
Convery et al. [56]	Static	–	–	✓	–	L2–J1–P1–SM
Major et al. [57]	Dynamic-automatic	2.3	0 to 14	✓	–	L2–J1–P1–DA
Cappa et al. [58]	Dynamic-automatic	–	− 7 to 15	–	✓	L3–J1–P2–DA
Novacheck et al. [59]	Dynamic-manual	–	− 20 to 20	✓	✓	L2–J1–P1–DM
Faustini et al. [60]	Static	–	–	✓	–	L2–J1–P1–SM
Bregman et al. [45]	Dynamic-manual	–	− 10 to 20	✓	✓	L3–J2–P1–DM
Ringleb et al. [61]	Dynamic-automatic	0.5	− 10 to 9	–	✓	L3–J1–P2–DA
Bielby et al. [62]	Dynamic-manual	–	− 50 to 20	✓	✓	L3–J1–P2–DM
Kobayashi et al. 2010 [63]	Dynamic-automatic	10	− 15 to 15	–	–	L3–J1–P1–DA
Lai et al. [64]	Dynamic-automatic	–	− 15 to 20	–	✓	L3–J1–P2–DA
Takahashi and Stanhope [65]	Dynamic-manual	–	0 to 10	–	–	L2–J1–P1–DM
Gao et al. [66]	Dynamic-automatic	10	− 20 to 15	✓	–	L3–J1–P1–DA
Schrank et al. [67]	Static	–	0 to 20	–	✓	L3–J1–P1–SM
Harper et al. [68]	Dynamic-automatic	–	–	–	–	L2–J1–P1–DA
Walbran et al. [44]	Static	–	0 to 20	–	–	L2–J1–P1–SM
Grigas et al. [69]	Dynamic-automatic	–	–	✓	–	L2–J2–P1–DA
Sheehan and Figgins [70]	Dynamic-manual	–	–	–	–	L3–J1–P1–DM
Chung et al. [43]	Dynamic-automatic	30	− 20 to 10	✓	–	L1–J1–P1–DA
Ielapi et al. [37]	Dynamic-automatic	1	− 25 to 25	✓	✓	L3–J1–P1–DA
Wach et al. [71]	Static	–	–	✓	–	L3-J1–P1–SA
Böhm et al. [72]	Dynamic-manual	–	–	✓	–	L1–J2–P1–DM
Totahet al. [36]	Dynamic-automatic	up to 100	− 25 to 25	✓	✓	L3–J1–P1–DA
Yamamoto et al. [73]	Dynamic-automatic	10	− 20 to 20	✓	–	L2–J1–P1–DA
Ashcraft et al. [74]	Dynamic-manual	–	− 10 to 5	–	–	L2–J1–P1–DM
Go et al. [75]	Static	–	− 8 to 8	–	–	L3–J1–P1–SM
Ramezani et al. [46]	Dynamic-manual	–	0 to 21	–	✓	L3–J1–P1–DM
Rogati et al. [76]	Dynamic-automatic	15	− 15 to 15	✓	✓	L3–J1–P1–DA
Shuman et al. [77]	Dynamic-automatic	0.75	− 3 to 20	✓	✓	L3–J1–P1–DA
Nigro et al. [78]	Dynamic-automatic	<1	0 to 15	✓	–	L3–J1–P1–DA
Anbuvalanraj et al. [79]	Static	–	0 to 5	–	–	L2–J1–P1–SA
Chatzistergos et al. [80]	Dynamic-automatic	0.017	2.6 to 6.8	–	–	L3–J1–P1–DA
Delbruel et al. [81]	Static	–	–	–	–	L2–J1–P1–SM
Khaing et al. [82]	Dynamic-automatic	–	− 10 to 10	✓	–	L2–J1–P1–DA
Rong et al. [83]	Static	–	0 to 7	–	–	L2–J1–P1–SA
Yeh et al. [84]	Dynamic-automatic	–	–	–	–	L3–J1–P1–DA
Bywater et al. [85]	Dynamic-automatic	4	− 15 to 15	–	–	L2–J1–P1–DA
Krummenacker et al. [86]	Static	–	–	–	–	L2–J1–P1–SM

See Fig. 8 for classifications

### In-situ versus benchtop testing

One way to categorize AFO stiffness measurement methods is whether the AFO is tested while worn by a human subject or secured to a testing apparatus. We will refer to these approaches as in-situ and benchtop methods, respectively. In-situ testing methods, also called “functional testing methods" [42], may flex the AFO through a load applied by the user’s biological limb or by an external load source while the foot and shank muscles are relaxed. Examples of external load sources include a plate rotated by an experimenter [87] or an isokinetic dynomometer like the Biodex [43]. The external loads facilitate passive ankle motion in order to minimize muscle force contributions when measuring the AFO forces. In-situ testing evaluates AFO properties under realistic use conditions, but limb-device interactions may introduce inconsistencies and make it difficult to isolate AFO properties from tissue mechanics.

Benchtop testing methods are the more common approach to measuring AFO stiffness. These methods often use a surrogate limb to secure the AFO and/or provide a contact surface for applying loads to deflect the AFO. Across benchtop testing methods, the design of the surrogate limb varies. They are made from a range of materials, including metal (Fig. 2B), wood (Fig. 2C), or plaster (Fig. 2E). Some use a single surrogate limb that is biologically accurate in appearance, often made using a mold of an AFO user’s lower limb. Another approach is designing a modular surrogate limb that uses interchangeable contact parts to test a wider range of AFO sizes. For example, the Bi-articular Reciprocating Universal Compliance Estimator (BRUCE) device [45] has multiple metal surrogate feet of varying sizes that can be switched out to match the foot length of the AFO. Similarly, the Stiffness Measurement Apparatus (SMApp) [36] mimics different calf sizes using custom, 3-D printed rings attached with a linear bearing on a metal surrogate shank. Benchtop testing methods effectively isolate the mechanical properties of AFOs and can be actuated with motors to provide consistent flexion speeds and ankle ranges of motion. However, these methods are limited by their reliance on biological approximations of ankle motion, which may not fully replicate the dynamics of the lower limb, potentially causing a mismatch between measured properties and what is experienced by the user.

### Operation and dynamics of testing methods

AFO testing methods vary in their operation and dynamics, affecting the type of data they measure. Static testing methods collect discrete data points at specific ankle angles. These methods often constrain the shank segment and calf cuff of the AFO, then hang weights from the AFO’s footplate (Fig. 2E) [9, 60]. Static testing methods can also be pulley-based, controlled by suspended masses [52] or a socket ratchet and crank arm system [55]. However, these methods cannot capture the hysteretic and viscoelastic properties of AFOs, as these characteristics are time-dependent and require continuous motion. In contrast, dynamic testing methods continuously operate through a range of motion to collect a time series of data (Fig. 2A–D). These methods can be manually actuated through human input [59, 65, 70, 87], or automatically using a motor [36, 37, 66, 77].

The testing method operation also affects experimental dynamics like flexion speed and range of motion. Dynamic testing methods have a range of flexion speeds depending on the actuation type. Typically, a method that uses automatic actuation operates at a flexion speed proportional to the power of the motor used. Manually operated methods usually do not have a precise, quantifiable set flexion speed due to variability from human operation. Flexion range and direction also varies between testing methods. Some operate through both plantarflexion and dorsiflexion, while others only move in one flexion direction. Peak ankle angles during gait are approximately 20 degrees in plantarflexion and 15 degrees in dorsiflexion [88]. Several testing methods can operate through this full range of motion [36, 37, 54, 59, 62, 66]. However, most methods are more limited and only operate through a smaller range, some as little as five degrees [52, 79].

### Sagittal plane testing

The overwhelming majority of AFO testing methods are designed to simulate AFO deflection in the sagittal plane, modeling ankle joint motion as a one degree of freedom hinge joint. As discussed in Sect. 2.1, this is a simplification of the ankle joint’s three degrees of freedom. Some AFOs, like solid ankle AFOs, are designed to stabilize lateral motion in the frontal plane. While the majority of gait motion occurs in the sagittal plane, frontal and transverse plane forces are still relevant.

The Evaluating Mechanical Properties In Rotating Exoskeletons (EMPIRE) device is unique in that it combines uniplanar motion with multiplanar force measurement. The EMPIRE flexes AFOs in the sagittal plane only while using a six-axis load cell to record the forces applied in all three anatomical planes [77]. The use of a multiaxial load cell captures the out-of-plane (transverse and frontal plane) forces when a sagittal-plane deflection is applied. However, deflection in the other planes is needed to capture multidimensional AFO stiffness.

### Multiplanar testing

The authors are aware of only five testing methods that deflect AFOs in multiple planes [48, 52, 58, 61, 62]. Their operation methods vary between in-situ [48], benchtop static [52], benchtop dynamic-automatic [58, 61], and benchtop dynamic-manual [62]. These multiplanar methods measure more all-encompassing data than single-plane methods, but also introduce certain limitations.

The major advantage of multiplanar methods is their ability to measure AFO properties under conditions that better represent the various loads experienced during walking. The details of the multiplanar testing characteristics vary between methods. Two of these methods only apply AFO flexion and measure properties in two planes, representing plantarflexion/dorsiflexion and inversion/eversion [48, 61]. Two other methods also only flex the AFO through plantarflexion/dorsiflexion and inversion/eversion, but account for internal/external properties by measuring the torques induced in that plane [52] or implementing a static shift in internal/external rotation between trials [58]. The most comprehensive multiplanar method is capable of applying a torque in all three anatomical planes, either in an isolated plane or combinations of the possible ranges of motion [62]. This variety in operation methods and planes tested demonstrates that multiplanar methods are highly versatile and can be tailored for specific AFO testing applications.

**Fig. 9:**
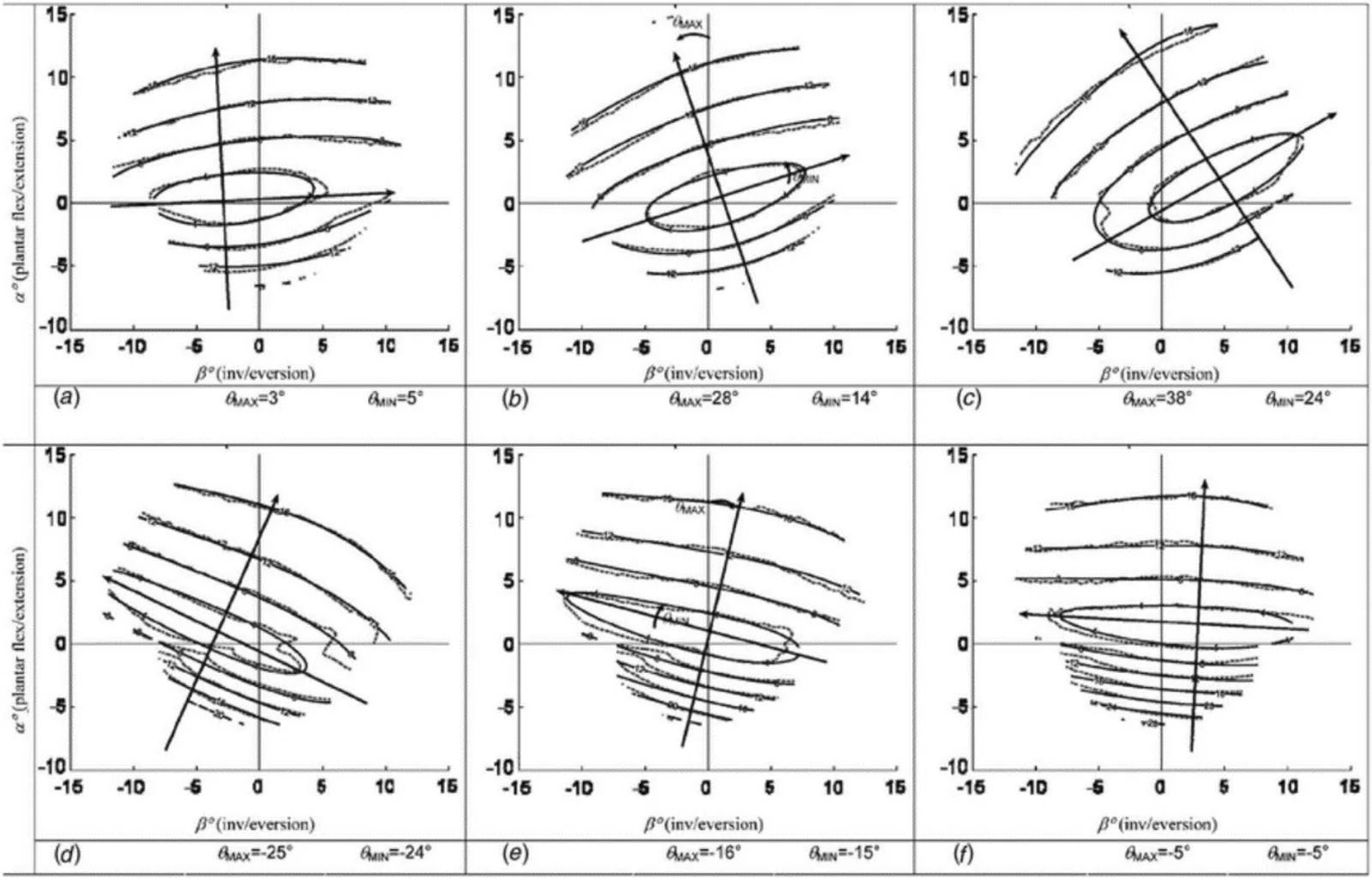
Multiplanar torque contour graphs, used with permission of American Society of Mechanical Engineers, from [58]; permission conveyed through Copyright Clearance Center, Inc

Despite these advantages, multiplanar methods also introduce limitations that must be acknowledged. Some multiplanar methods come with individual drawbacks. For example, one method secures AFOs using three holes drilled into the AFO footplate, causing permanent deformation that make the AFO clinically unusable [52]. Another method is manually operated, which improves its accessibility, but likely causes inconsistencies in testing speed that might affect measurements [62]. A more general limitation of multiplanar methods is difficulty representing their measurements into clinically relevant parameters. One method uses contour plots to represent the data, illustrating the total moment measured from various combinations of AFO angular displacements [58] (Fig. 9). These contour plots provide comprehensive measurements with instructions for interpreting, but are difficult to summarize into a representative parameter. Other methods represent multiplanar stiffness using an individual measurement for each plane [48, 62] or direction [61]. Reporting three to four stiffness values for a single AFO is informative, but the burdens of data complexity and the challenge to interpret the overall AFO stiffness might outweigh the benefits.

### Multi-joint testing

Most methods test the ankle joint while assuming a rigid footplate. However, the BRUCE, a manually-operated dynamic testing device, stands out from other sagittal plane testing methods because it models the metatarsophalangeal (MTP) joint in addition to the ankle joint [45]. The MTP joint connects the bones of the foot to the bones of the toes, which the BRUCE represents as a hinge joint that is manually actuated. A force plate collects the resulting AFO reaction forces to measure the AFO’s footplate stiffness. By including the MTP joint, the BRUCE is capable of collecting more extensive stiffness measurements of the entire AFO.

## Discussion and design challenges

Significant advancements in AFO stiffness measurement have improved our understanding of AFO mechanical properties. These advancements have also enabled work evaluating the effect of different design and testing parameters on measured AFO properties, and how AFO stiffness influences user outcomes. Despite this progress, there is a lack of between-method reliability, underscoring the need for developing standardized testing protocols and terminology. In this work, we categorized all available AFO testing methods based on key properties and created a taxonomy scheme (Fig. 8) based on limb presence, joints tested, planes of motion, static vs dynamic motion, and type of operation (automatic vs manual). This taxonomy allows quick categorization and comparison of specifications across testing methods (Table 1).

Key elements of benchtop testing that require further investigation and would benefit from standardization include the bending axis location, testing planes, speed, and range of motion. Other challenges include standardizing in-situ testing, measurement validation, developing clinically-relevant models of AFOs, and creating frameworks for clinical application. The following sections expand on each of these topics.

### Bending axis location and design

AFO benchtop testing methods often assume a fixed location of the bending axis or surrogate ankle. However, the AFO’s center of rotation might not align with that of the testing apparatus, potentially influencing the measured stiffness. Research groups have developed various approaches to this challenge. Some groups define specific rotation axis locations for standard AFO sizes based on anthropometry and medical imaging data, then position the testing method’s rotation axis accordingly for different AFO sizes [77]. Another proposed method accounts for misalignment in post-processing by matching the AFO’s and testing method’s rotation axis using geometric relationships [36], enabling a fixed surrogate ankle for all AFO sizes, but this has not been experimentally validated. In contrast, some studies do not specify alignment procedures, and for in-situ testing, alignment is naturally achieved through proper AFO fit on the user.

It remains unclear whether ankle location mismatch is a significant source of error in AFO stiffness measurement. Research has shown that anterior-posterior AFO misalignment results in significantly higher stiffness than center alignment [66], and that proximal-distal shifts in AFO bending axis influence running mechanics [89]. These findings suggest that AFO rotation axis location is a key factor and should be standardized to ensure consistent, accurate measurements. We recommend developing standard rotation axis locations for AFO testing methods, corresponding to various male and female AFO sizes. This would likely improve inter-method reliability, and measure AFO properties that better represent the user experience. Furthermore, since the biological ankle translates during walking [31], a dynamically translating ankle joint model might enable more representative measurements. The effect of biologically relevant translations in rotation axis location should be evaluated to inform standardization of the ankle joint model and determine whether a dynamically shifting joint is necessary.

### Planes of motion

Another complexity of the ankle joint is that it has three rotational degrees of freedom but is often conceptualized as a hinge joint. Most benchtop testing methods flex AFOs in the sagittal plane only, but multiplanar testing more accurately replicates biological gait. To fully realize the potential of multiplanar AFO measurement, more advanced motion and force sensing could be implemented through embedded sensors or motion capture based methods. This would enable multidimensional matrices quantifying AFO properties.

Although some benchtop testing methods do measure AFO properties in multiple planes, they introduce challenges in reporting stiffness and ensuring accessibility. Representations of multiplanar data can be difficult to interpret, and the standard approach should report a stiffness in each plane to balance comprehensive measurement with clinical utility. Additionally, multiplanar systems are typically significantly more complex and expensive than single-plane methods, limiting their accessibility. Although high quality, meticulously conducted research is essential for advancing the field, it might not be feasible to implement these systems in widespread clinical settings. The most accurate testing is desired for improved treatment outcomes, but retaining affordability for clinical applications is vital to ensure optimal AFO prescriptions for users. Therefore, standardization should focus on simpler, more cost-effective sagittal-plane methods using dynamic operation to better represent gait.

### Testing speed and range of motion

There has been a noticeable shift from static to dynamic testing of AFOs, which has improved AFO mechanical modeling by capturing hysteresis and measuring stiffness for a range of angles. Key operational choices of dynamic testing methods that should be standardized include testing speed and range of motion. Many testing methods are limited by slower speeds and smaller ranges of motion than the ankle experiences during gait. Furthermore, testing methods operate at a set speed between two prescribed angles, but ankle angular velocity is nonlinear and the gait cycle has four distinct ankle angle peaks [88]. These operational limitations may result in data that is not representative of the user experience or the complete scope of functional gait.

To address this, a method for deflecting AFOs through a nonlinear ankle angle trajectory should be developed to evaluate the effect of dynamically changing rotational speed on measured stiffness. This would inform how closely testing methods need to mimic functional gait to provide accurate data. We recommend operating benchtop testing devices at gait-representative speeds and through physiologically relevant angles, while considering the AFO’s intended use. For example, an AFO designed to limit a user’s range of motion could be tested through angles slightly larger than those expected during its use, rather than through the full range of motion of an unimpaired gait cycle. This would provide information about the forces used to limit motion and what muscle forces are needed to overcome it, without over-flexing the AFO and causing damage.

### Accommodating different AFO sizes and designs

Another crucial factor for ensuring clinical relevance is developing methods that can test AFOs of varying sizes and designs. Many off-the-shelf AFOs are available in four to five sizes ranging from XS to XL, and pediatric AFOs introduce an even broader size range. A recent study found that both AFO ankle and footplate stiffness increase with AFO size, but that the effect of size varies across AFO types [90]. Therefore, measuring the properties of a single AFO is insufficient to represent a product. Instead, the size must be reported, and multiple sizes should be evaluated to capture the full range of mechanical properties within a given design. This underscores the need for testing methods to accommodate varied sizes to provide a comprehensive evaluation of AFO properties. Relevant testing method design considerations include footplate clamp length, footplate clamp width, shank height, and shank width, which may be addressed by accommodating the full range of sizes or implementing modular components.

Besides sizing differences in both adult and pediatric populations, many current methods are designed to interface with posterior leaf spring or similar AFOs. However, there are other AFO types with anterior or medial/lateral components, where quantifying multiplanar properties is particularly relevant (Sect. 4.4). New innovative AFO designs exist and are expected to develop further in the future, so measurement methods should be capable of testing these novel devices. Additionally, custom AFOs can have unique geometry depending on the user’s anatomy, and testing methods should accommodate this variability. Introducing modular testing clamps with minimally intrusive, easily interchangeable components would address these complexities by enabling adaptable contact parts tailored to different AFO geometries. Existing clamping methods often use traditional metal materials, but incorporating more advanced components with flexible materials that conform to shape would enable a secure fit for any AFO design in a single clamp. Also, a standard categorization of AFO types would facilitate the design of more inclusive mechanical testing methods. Considering unconventional and diverse AFO designs would ensure widespread clinical accessibility of AFO testing for a variety of pediatric and adult AFO users. In terms of measurements, novel AFO designs with dampers, springs, or clutch mechanisms should quantify viscoelastic properties and energy storage capabilities, best represented in dynamic higher-order models relating ankle torque and angle.

### Standardizing in-situ methods

In-situ measurement methods have the advantage of testing AFOs on a user’s limb while flexing through natural biological movements. However, factors like the limb-device interface and soft tissue interactions are not well-characterized, causing difficulties isolating AFO properties from limb properties. While using a biological limb is beneficial for representing real-world use, individual anatomical differences mean these results are likely most applicable to custom AFOs but might not be repeatable or generalizable between users. A possible direction for the further development of in-situ testing is to implement additional, more advanced sensing like motion capture and force/pressure sensing at the tissue-device interface. Video-based or sensor-based motion capture would enable multi-dimensional motion models for evaluating the AFO’s dynamic functional performance.

To our knowledge, there has been no direct comparison between in-situ and benchtop methods to evaluate the effects of their differences. A comparison is needed to better understand each method’s strengths and limitations, and unveil tissue-device interactions. Benchtop methods likely enable more controlled and repeatable testing, but may be missing key mechanics that occur when the device is used with a biological limb.

### Measurement validation

A source of difficulty for all AFO testing methods is the validation of stiffness measurements. Research groups often test the reliability of their method with factors like different operators or testing sessions, which is important for ensuring consistency within a method. However, validation remains difficult because there is no standard AFO stiffness measurement to check against. Some research groups have attempted to address this challenge by conducting cross-comparisons between methods [9, 36], but this is not true validation.

One potential solution we suggest is the introduction of standard, physical AFO stiffness models that can be used to validate dynamic stiffness measurement in multiple planes. Alternatively, developers of testing methods could create their own custom validation AFOs with spring components of known stiffness, for example. These could be an effective temporary solution for improved comparisons between testing methods until governing bodies or organizations, e.g. the International Society for Prosthetics and Orthotics, introduce standardized methods for testing and reporting error. The taxonomy we introduced to organize and classify testing methods by design features and operational properties enables direct cross-method comparisons and could inform measurement validation. Our standard classification system can guide which methods can be directly compared, and specific validation models could be developed for each category.

### Creating clinically-relevant, high-fidelity models

As high-accuracy testing methods are developed, we need a way to describe the complex behaviors of AFOs. An important consideration is striking the balance between accessibility and accuracy. Simple descriptors are necessary for clinical use and cross-study comparison, but there is also a need for high-order, complex dynamic models that fully describe AFO properties. Some research groups have begun to address this by considering additional AFO properties like damping [35], hysteresis [36], and neutral angle [45]. A piece-wise linear stiffness model has been used to describe AFO stiffness in both plantarflexion and dorsiflexion [36, 37].

However, these models do not fully capture AFOs’ key viscoelastic or energy storage characteristics. Higher-order models with multiple parameters are needed to capture these dynamic behaviors under multiple conditions. These models should incorporate flexion angle, speed, and direction for a higher-fidelity system identification of AFO properties. This complex framework could then be distilled into clinically-relevant interpretations by describing sagittal-plane stiffness at discrete increments of other testing properties, for example.

High-fidelity AFO modeling is also needed for computational simulation. Several computational methods for evaluating AFO properties have been developed, detailed in a literature review by Ielapi et al. [27]. These models can be improved and validated once accurate, experimental data is available to reflect real-world conditions.

### Clinical applications

Finally, translating these mechanical testing methods and their results into clinical practice is the essential next step and should be done through evidence-based protocols. Possible protocol structures could be modeled after proposed frameworks that evaluate energy cost and walking speed across multiple AFO stiffness levels [25], or that make iterative adjustments based on specific gait assessment metrics [26]. Ultimately, accurate mechanical testing of AFO properties is essential for enabling generalizability of clinical AFO testing. AFO studies that include both detailed user information (e.g. demographics, gait performance metrics, activity levels, and preferences) along with the AFO properties (e.g. design, shape and trimlines, thickness, material, stiffness) would allow a much clearer picture of the impact of AFO properties on the user. This is necessary for the development of standardized protocols and practical decision algorithms to guide clinician decision making, helping bridge the gap between engineering testing and clinical implementation.

### Scoping review limitations

This scoping review was limited to analysis of published AFO testing methods. Other groups may have worked on this topic without publication, especially in clinical settings. Additionally, some included papers had incomplete reporting of parameters (e.g., flexion speed), limiting direct comparisons of all factors. We utilized all available parameters to create as detailed a categorization as possible.

## Conclusion

Valuable progress has been made in understanding AFO mechanical properties and developing AFO testing methods, but these methods are not standardized and come with various design challenges. In this work, we define a taxonomy to classify testing methods using standard categories based on testing method design and operation specifications. This common language will enable inter-method comparisons by providing a consistent framework for evaluating various AFO testing methods. Future work should focus on developing standardized, repeatable methods for biomimetic measurement of AFO mechanical properties to ensure they accurately reflect the conditions experienced by users. Additionally, establishing standard validation protocols is critical to ensure measurement accuracy and inter-method reliability. These methods must remain practical and affordable, enabling accurate measurement of AFO properties in a variety of clinical and research settings. This work is clinically significant because understanding AFO properties is essential for improving their design, informing prescription practices, and ultimately optimizing user outcomes and quality of life.

## Data Availability

No datasets were generated or analysed during the current study.

## Abbreviations

AFO: Ankle-foot orthosis
EMPIRE: Evaluating mechanical properties in rotating exoskeletons
SMApp: Stiffness measurement apparatus
KST: Kentucky stiffness tester
BRUCE: Bi-articular reciprocating universal compliance estimator
MTP: Metatarsophalangeal
